# Multicenter development and external validation of clinical–radiomics models to predict surgically confirmed upstaging in biopsy‐proven DCIS using DCE‐MRI

**DOI:** 10.1002/acm2.70637

**Published:** 2026-05-31

**Authors:** Xi Hu, Lujie Qian, Jie He, Beili Shou, Ying Hu, Qingqing Chen, Enhui Xin, Fei Li, Zongyu Xie, Yue Qian, Feifei Lou, Nan Liu, Yu Kuang, Hongjie Hu

**Affiliations:** ^1^ Department of Radiology Sir Run Run Shaw Hospital Zhejiang University School of Medicine Hangzhou China; ^2^ Department of Magnetic Resonance Imaging The First Affiliated Hospital of Zhengzhou University Zhengzhou China; ^3^ Department of Research and Development Shanghai United Imaging Healthcare Co. Ltd Shanghai China; ^4^ Department of Radiology The First Affiliated Hospital of Bengbu Medical University Bengbu China; ^5^ Department of Translational Medicine Sir Run Run Shaw Hospital Zhejiang University School of Medicine Hangzhou China; ^6^ Department of Radiation Oncology University of South Florida Morsani College of Medicine Tampa Florida USA; ^7^ Tampa General Hospital Cancer Institute Tampa Florida USA

**Keywords:** breast MRI, DCIS upgrade prediction, multicenter external validation, radiomics

## Abstract

**Purpose:**

Ductal carcinoma in situ (DCIS) diagnosed on core biopsy is frequently upgraded to invasive carcinoma at surgery, which may change indications for sentinel lymph node biopsy. Routine breast MRI has limited ability to detect occult invasion preoperatively. This study aimed to develop and externally validate an MRI‐based model combining clinical variables, conventional MRI findings, and dynamic contrast‐enhanced (DCE) MRI radiomics to predict invasive upgrade in biopsy‐proven DCIS.

**Methods:**

This retrospective multicenter study enrolled 478 patients from three hospitals (2014–2019). Center 1 contributed 314 patients, randomly split into a training set (*n* = 251) and an internal test set (*n* = 63); Centers 2 (*n* = 39) and 3 (*n* = 62) formed two independent external test sets. Radiologists assessed conventional MRI features, including lesion size, enhancement descriptors, and diffusion‐derived apparent diffusion coefficient metrics. Tumors were segmented on DCE MRI. Radiomics features with intraclass correlation coefficient > 0.85 were *z*‐score normalized, selected using least absolute shrinkage and selection operator regression, and used to train multiple machine learning classifiers; the best‐performing model generated a radiomics score. Model selection and hyperparameter tuning were performed by cross‐validation within the training set only. Clinico‐radiologic, radiomics, and combined models were evaluated using receiver operating characteristic (ROC) curve analysis, calibration, and decision curve analysis, the area under the curve (AUC) was calculated.

**Results:**

Six clinico‐radiologic factors and 13 radiomic features were retained. In the two external test sets, the clinico‐radiologic, radiomics, and combined models achieved AUCs of 0.61 (95% CI, 0.43–0.79) and 0.71 (0.58–0.83), 0.70 (0.54–0.86) and 0.71 (0.58–0.84), and 0.76 (0.60–0.91) and 0.77 (0.65–0.89), respectively. The combined model provided the highest net benefit on decision curve analysis.

**Conclusion:**

A combined clinico‐radiologic and DCE‐MRI radiomics model showed multicenter, externally validated performance for preoperative prediction of invasive upgrade in DCIS, supporting risk stratification for surgical planning.

**Abbreviations**
ADC: apparent diffusion coefficient
AUC: area under the curve
CI: confidence interval
DCA: decision curve analysis
DCE: dynamic contrast‐enhanced
DCIS: ductal carcinoma in situ
DT: decision tree
DWI: diffusion‐weighted imaging
ICCs: intraclass correlation coefficients
ICs: invasive components
IDI: integrated discrimination improvement
LASSO: least absolute shrinkage and selection operator
LR: logistic regression
MRI: magnetic resonance imaging
NME: non‐mass enhancement
OR: odds ratio
RF: random fores
ROC: receiver operating characteristic
SLNB: sentinel lymph node biopsy
SVM: support vector machinet
T1WI: T1‐weighted imaging

## 1 INTRODUCTION

Ductal carcinoma in situ (DCIS) currently accounts for approximately one‐fifth of all newly diagnosed breast cancer cases.^1^, ^2^ The incidence of DCIS has notably increased in recent years, reflecting advancements in breast cancer screening techniques.^3^, ^4^ While DCIS itself is not fatal, the majority of cases do not progress to invasive carcinoma.^5^, ^6^ As a result, there has been a growing interest in evaluating the outcomes of active surveillance rather than immediate surgical intervention, to avoid potential overdiagnosis and unnecessary treatments.^7^, ^8^, ^9^

Emerging evidence suggests that active surveillance of DCIS, as opposed to surgical treatment, offers similar survival outcomes and may be a safe alternative.^10^, ^11^, ^12^ A critical factor in implementing this approach effectively is the identification of “invasive components” within DCIS, which refer to areas of invasive cancer that may be present within or adjacent to the in situ lesions but are not detectable at the time of diagnosis. These components, both clinically and pathologically, are important because their presence could indicate a higher risk for progression to invasive disease. Pathologically, they are characterized by the infiltration of cancer cells beyond the basement membrane of the ductal structures, while clinically, they may be associated with features such as lymphovascular invasion, higher grade lesions, or atypical histological patterns.

In addition, DCIS is theoretically non‐invasive and therefore does not metastasize to the axillary lymph nodes.^13^ The guideline from the National Comprehensive Cancer Network indicates that sentinel lymph node biopsy (SLNB) is generally not advised for most DCIS lesions when planning surgical intervention. However, in situations where DCIS includes invasive components (ICs), axillary surgery is crucial.^14^, ^15^, ^16^ DCIS upstages to invasive ductal carcinoma in approximately 25%–50% of cases during breast procedures.^17^, ^18^, ^19^, ^20^, ^21^, ^22^ Therefore, improving the ability to predict whether women with DCIS have occult ICs has important clinical implications.

Women diagnosed with breast cancer often undergo breast magnetic resonance imaging (MRI) for preoperative evaluation to determine disease extent, detect multifocality, and guide surgical planning. However, in patients with biopsy‐confirmed DCIS, preoperative breast MRI is not reliable in predicting occult ICs because conventional imaging features (e.g., enhancement patterns, lesion morphology) frequently overlap between pure DCIS and lesions with microinvasion, leading to limited specificity and sensitivity.^23^, ^24^, ^25^ Recently, radiomics emerged as a burgeoning research area, which can transform medical images into high‐throughput quantitative features.^26^, ^27^ Some previous studies have attempted to use radiomic analysis to predict whether DCIS contains ICs.^28^, ^29^, ^30^, ^31^ However, these studies suffered from a small sample size ^28^, ^29^, ^31^, no external validation,^28^, ^30^, ^31^ and a lack of conventional MRI quantitative parameters.^28^, ^29^, ^30^, ^31^ To our knowledge, this study represents the most extensive sample size of related studies to date based on MRI radiomics features; moreover, it involved two external validation sets.

This study aimed to evaluate whether radiomics features from dynamic contrast‐enhanced (DCE) MRI can preoperatively predict surgically confirmed upstaging in DCIS and to compare their performance with conventional imaging features (i.e., qualitative and semi‐quantitative descriptors defined by the BI‐RADS lexicon, such as lesion type, margins, internal enhancement characteristics, and kinetic curve patterns).

## 2 METHODS

### 2.1 Patients

This retrospective analysis was approved by three ethics committees, and the need for informed consent was waived.

**FIGURE 1:**
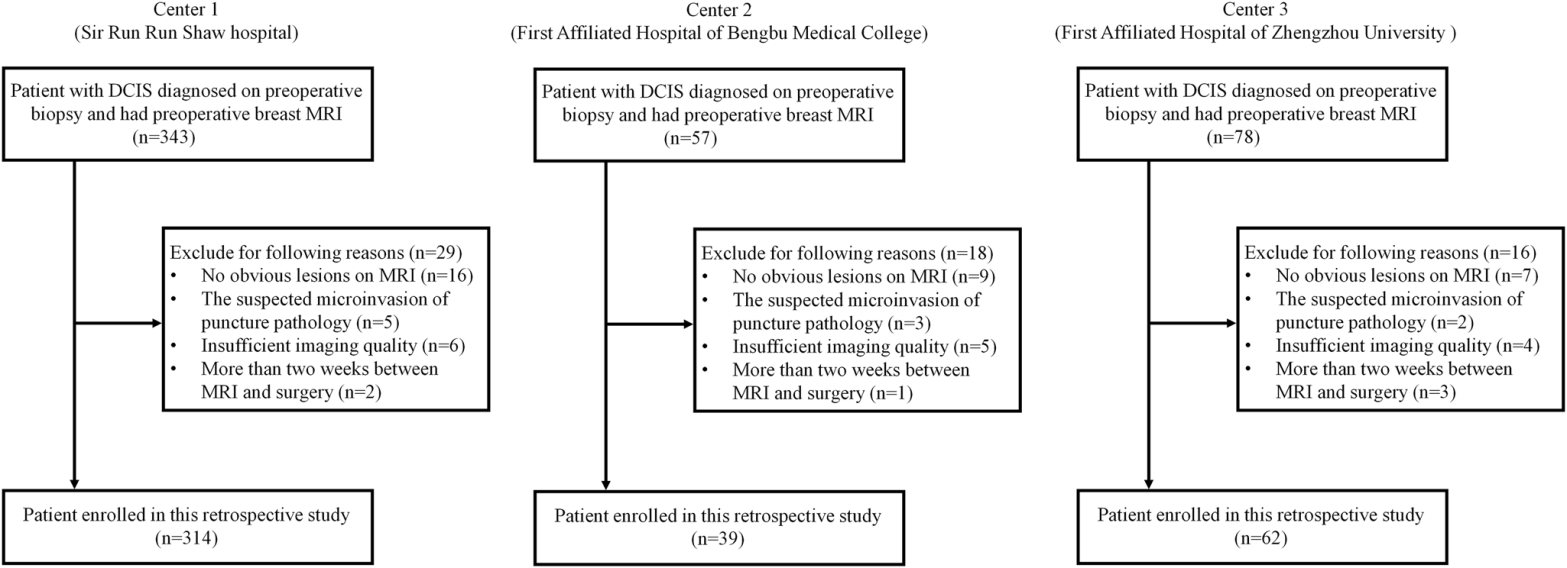
Recruitment of patients.

Individuals with DCIS who received a biopsy diagnosis and who had preoperative MRI between January 1, 2014, and December 31, 2019, at Center 1 (Sir Run Run Shaw Hospital, Zhejiang University School of Medicine) were recruited from the pathology database. Inclusion criteria were: (1) histologically confirmed DCIS via core‐needle biopsy; (2) availability of preoperative breast MRI including DCE and DWI sequences; (3) subsequent surgical excision with definitive pathology. Exclusion criteria were: (1) No obvious lesions on MRI; (2) The suspected microinvasion of puncture pathology; (3) Insufficient imaging quality; (4) More than two weeks between MRI and surgery. A total of 343 individuals were initially enrolled in this analysis. In addition, following the same criteria for inclusion, we also initially recruited 57 individuals from Center 2 (The First Affiliated Hospital of Bengbu Medical University) and 78 individuals from Center 3 (The First Affiliated Hospital of Zhengzhou University). A detailed list of inclusion and exclusion criteria can be found in Figure 1. Finally, the study included 314 patients from Center 1 (primary cohort), 39 from Center 2, and 62 from Center 3.

The patient data, including age, bloody nipple discharge and postoperative pathological results, were derived from electronic medical clerking.

### 2.2 MRI data acquisition

For the three hospitals, all breast MRI scans were performed using either a 3.0 T or 1.5 T scanner. Imaging sequences included DCE MRI and diffusion‐weighted imaging (DWI). During scanning, axial DWI images were acquired with two different *b*‐values (0 and 800 or 1000 sec/mm^2^) before the contrast medium was injected. The apparent diffusion coefficient (ADC) maps were obtained using the monoexponential model in our workstation. A pre‐scan of fat‐suppressed T1‐weighted imaging (T1WI) was performed before scanning multi‐phase dynamic enhanced images. After the administration of the gadolinium contrast agent, fat‐suppressed T1WI images were acquired roughly every 60 s, for a total of 6–8 acquisitions. Detailed MRI scanning parameters can be found in the Supplementary Methods section and Table S1.

### 2.3 Radiographic evaluation

Two radiologists (Reader 1, F.L., 10 years of breast imaging experience.; Reader 2, J.H., 9 years of breast imaging experience) assessed all MR images without knowledge of the clinical and pathologic information. Both radiographic observers were members of the institution's breast multidisciplinary team and had interpreted > 1000 breast MRI examinations. Before the research, they spent a full day reviewing relevant knowledge and practical guidance detailed in the fifth edition of the American College of Radiology BI‐RADS lexicon. Eighty cases of breast cancer randomly chosen from the breast cancer database underwent individual review, followed by a group review. The senior physician (H.H., 20 years of breast imaging experience) who coordinated and oversaw the training program, affirmed that the doctors met the study's criteria.

The evaluation of routine imaging features mainly referred to Lee et al.,^32^ Bickel et al.,^33^ and BI‐RADS lexicon and included 15 major imaging features: (1) tumor long diameter; (2) tumor volume^34^; (3) quadrant with tumor involvement (0, one quadrant involved; 1, more than one quadrant involved); (4) signal intensity on T2 weighted images (0, iso and low; 1, high); (5) mass or non‐mass enhancement (NME) (0, NME; 1, mass); (6) enhancement pattern (0, homogeneous and clumped; 1, heterogeneous and rim); (7) intratumor necrosis (0, absent; 1, present); (8) ductal ectasia (0, absent; 1, present); (9) nipple‐areolar complex invasion (0, absent; 1, present); (10) initial enhancement pattern of dynamic curve (0, slow and medium; 1, fast); (11) washout during the delayed phase (0, absent; 1, present); (12) mean ADC (ADCmean); (13) maximum measurable ADCmean area; (14) minimum ADC (ADCmin); (15) maximum measurable ADCmin area. Readers 1 and 2 jointly evaluated the conventional image features. Upon any disagreement, they discussed until they reached an agreement.

### 2.4 Tumor segmentation and radiomics feature extraction

Reader 2 utilized the open‐source software ITK‐SNAP (version 4.0, www.itksnap.org) to carry out segmentation of the volume of interest for all patient lesions. Contours were drawn slice‐by‐slice on the second post‐contrast DCE series, including all contiguous enhancing voxels corresponding to the index lesion and excluding adjacent parenchyma/fat, vessels/ducts not involved, skin/chest wall, background enhancement, biopsy‐related artifacts, and clearly non‐enhancing intralesional components. For non‐mass enhancement, the volume of interest (VOI) followed the contiguous ductal/segmental pattern of the index lesion only.

The magnetic resonance images were firstly preprocessed: they were resampled to isotropic resolution (voxel size = 1×1×1 mm^3^) using B‐spine interpolation, followed by normalization as inputs for radiomic feature extraction. All images were processed through the uAI Research Portal (uRP; Shanghai United Imaging Intelligence, Co., Shanghai, China).^35^

Radiomic features were extracted using the uRP, which is embedded with PyRadiomics. For each image, three categories of features were initially extracted: 18 first‐order statistics, 14 shape‐based features, and 72 texture features. The texture features comprised 21 gray‐level co‐occurrence matrix (GLCM) features, 14 gray‐level dependence matrix (GLDM) features, 16 gray‐level run length matrix (GLRLM) features, 16 gray‐level size zone matrix (GLSZM) features, and 5 neighborhood gray‐tone difference matrix (NGTDM) features. Moreover, 24 image filters were applied, an additional 432 first‐order features and 1728 texture features were obtained. Thus, a total of 2264 radiomic features were derived from each subject. Supplementary Methods show the details of the radiomics features.

### 2.5 Intra‐ and interobserver agreement

To evaluate inter‐observer reproducibility, both readers independently re‐segmented 30 randomly selected cases one month after the initial segmentation. ICCs were calculated for all extracted features; only those with ICC > 0.85 (indicating excellent agreement) were retained for subsequent analysis. This stringent threshold minimizes the impact of segmentation variability on model performance.

### 2.6 Feature selection and model construction

In this step, we constructed three models (Clinico‐radiologic, Radiomics, and Combined models) to predict invasiveness.

In Center 1, the 314 patients were randomly allocated into two groups at a 4:1 ratio, resulting in a training set comprising 251 cases and an internal test set consisting of 63 cases. Cases from Centers 2 (39 cases) and 3 (62 cases) were used as the external test set.

Clinico‐radiologic model: Univariate analysis was performed in the training dataset to investigate the differences in the clinico‐radiologic features between the IC‐absent groups and the IC‐present groups. To account for multiple comparisons, *P* values from the univariate analyses were further adjusted using the Benjamini–Hochberg procedure to control the false discovery rate. Features that remained significant after BH correction were then entered into a multivariate logistic regression model to identify the key predictors of DCIS with ICs and to construct the clinico‐radiologic model.

Radiomics model: Firstly, the extracted features (ICCs > 0.85) were *Z*‐score normalized using the mean and standard deviation derived exclusively from the training set. This standardized training set was then subjected to least absolute shrinkage and selection operator (LASSO) regression with five‐fold cross‑validation to determine the optimal alpha value and identify the most predictive features prior to model construction. To maximize the recognition rate of the radiomics algorithm, various machine learning classifiers, such as bagging decision tree, decision tree (DT), logistic regression (LR), random forest (RF) and support vector machine (SVM) were applied to construct models using the selected features, and the predicted probability of the optimal model served as the radscore.

Combined model: A combined model was developed by integrating the selected clinico‐radiologic risk features with the radscore. LR was employed for this integration because of its interpretability, robustness, and ability to handle mixed data types. The final model was visualized as a nomogram to facilitate individualized prediction of invasive carcinoma in patients with DCIS.

Several strategies were implemented to mitigate overfitting. First, only features with excellent test‐retest reproducibility (intraclass correlation coefficient > 0.85) were retained. Second, LASSO regression with five‐fold cross‐validation was applied for dimensionality reduction within the training set. Third, all image preprocessing steps, including isotropic resampling (1 × 1 × 1 mm^3^) and *Z*‐score normalization, were performed using parameters derived exclusively from the training set and subsequently applied to the internal and external test sets. Finally, model performance was evaluated on strictly held‐out internal and external test sets that were not used in any stage of feature selection or model training.

### 2.7 Assessment of the performance of different models

To evaluate the ability of the model to predict ICs in DCIS, we calculated parameters such as the area under the curve (AUC) of the receiver operating characteristic (ROC) curve, sensitivity, specificity and accuracy between the training and test sets. The accuracy of the prediction results can be evaluated by comparing the predicted probabilities and the actual probabilities. The efficacy of the model was assessed through decision curve analysis (DCA), a method that evaluates the overall net gain of various threshold probabilities within the complete retrospective group.

### 2.8 Statistical analysis

Statistical analysis was performed using SPSS software version 26.0 (IBM, Armonk, NY, USA) and R software version 4.2.1 (R Foundation for Statistical Computing, Vienna, Austria). The normality of distribution was assessed for continuous variables using the Kolmogorov–Smirnov test. A *t*‐test was employed for comparing variables with a normal distribution. Otherwise, the Mann–Whitney U test was used. For categorical variables, the chi‐square test or Fisher's exact test was used. For evaluating the discrimination of each model, the AUC of the ROC curve was used. The optimal cutoff value from the training set, as determined by the Youden index, was used to calculate the diagnostic sensitivity, specificity and accuracy. Clinical utility was evaluated using DCA, and AUCs were compared between different models using the DeLong test. The performance improvement introduced by the combined model integrating clinico‐radiologic and radiomics features was quantified by integrated discrimination improvement (IDI).

## 3 Results

### 3.1 Basic clinico‐radiologic characteristics and construction of the clinical factor model

In Center 1, 155 (49.4%) of 314 patients had ICs in surgical pathology, in center 2, 15 (41.9%) of 39 patients had ICs in surgical pathology, and in Center 3, 29 (46.8%) of 62 patients had ICs on surgical pathology. Table 1 presents the clinical and MRI features of all patients in the three cohorts. After Benjamini–Hochberg correction for multiple comparisons, ductal ectasia was excluded from further analysis (Table S2). Multiple LR analysis further confirmed that six factors persisted as independent predictors among the clinical and radiologic factors (p < 0.05). A clinical history of bloody nipple discharge (odds ratio [OR], 3.977; 95% confidence interval [CI], 1.529–10.346), tumors with longer long diameter (OR, 1.834; 95% CI, 1.313–2.560), smaller tumor volume (OR, 0.937; 95% CI, 0.909–0.965), more than one quadrant involved (OR, 2.393; 95% CI, 1.069–5.354), larger maximum measurable ADCmean area (OR, 1.008; 95% CI, 1.001–1.015) or smaller ADCmin (OR, 0.042; 95% CI, 0.007–0.261) were likely to be DCIS with IC. The AUCs of the clinico‐radiologic model for the training set, the internal test set, and the two external test sets were 0.816, 0.746, 0.611, and 0.705, respectively.

**TABLE 1:** Clinical and histologic characteristics of all patients in the three cohorts.

	Center 1				Center 2				Center 3			
	All patients	Pure DCIS	DCIS with ICs		All patients	Pure DCIS	DCIS with ICs		All patients	Pure DCIS	DCIS with ICs	
Clinico‐radiologic features	(*N* = 314)	*N* = 159 (51%)	*N* = 155 (49%)	p^*^	(*N* = 39)	*N* = 16 (41%)	*N* = 23 (59%)	p^*^	(*N* = 62)	*N* = 35 (56%)	*N* = 27 (44%)	p^*^
Age (year)	48.45 (9.20)	47.70 (9.45)	49.21 (8.90)	0.145	49.7 (8.9)	48.2 (9.4)	50.8 (8.4)	0.368	49.3 (8.9)	50.1 (8.6)	48.2 (9.3)	0.398
Tumor long diameters (cm)	2.02 [1.31, 3.22]	1.53 [1.05, 2.42]	2.57 [1.80, 3.89]	<0.001	2.65 [1.70, 3.49]	2.08 [1.44, 2.74]	2.87 [2.02, 4.20]	0.043	2.66 [1.78, 4.30]	2.06 [1.35, 3.80]	3.48 [2.15, 5.23]	0.008
Tumor volume (cm^3^)	2.72 [0.90, 8.41]	1.32 [0.56, 4.14]	5.07 [2.28, 12.17]	<0.001	4.44 [1.79, 7.42]	2.51 [1.27, 5.57]	5.68 [2.98, 8.70]	0.009	4.12 [1.09, 9.59]	1.82 [0.81, 7.54]	7.14 [2.28, 11.33]	0.003
ADCmean (×10^−3^ mm^2^/s)	1.43 [1.24, 1.66]	1.45 [1.28, 1.74]	1.38 [1.20, 1.60]	0.003	1.45 [1.28, 1.61]	1.48 [1.32, 1.65]	1.42 [1.24, 1.58]	0.171	1.41 [1.22, 1.62]	1.38 [1.19, 1.58]	1.44 [1.26, 1.68]	0.091
Maximum measurable ADCmean area (mm^2^)	40.25 [20.60, 82.40]	25.90 [17.30, 54.75]	62.50 [33.10, 109.65]	<0.001	72.3 [36.2, 106.1]	45.4 [20.8, 88.0]	94.0 [69.0, 146.4]	0.008	49.40 [16.00, 116.60]	23.40 [15.00, 56.80]	76.50 [30.40, 91.60]	0.002
ADCmin (×10^−3^ mm^2^/s)	1.24 [1.06, 1.42]	1.32 [1.14, 1.57]	1.16 [1.02, 1.29]	<0.001	0.98 [0.84, 1.11]	0.96 [0.81, 1.10]	1.01 [0.87, 1.16]	0.284	1.14 [0.93, 1.29]	1.14 [0.93, 1.30]	1.13 [0.93, 1.29]	0.761
Maximum measurable ADCmin area (mm^2^)	8.20 [4.30, 14.10]	4.90 [4.30, 9.80]	9.40 [5.50, 16.50]	<0.001	9.5 [5.3, 14.2]	12.0 [7.9, 17.3]	7.6 [4.8, 11.2]	0.029	9.9 [5.2, 15.5]	12.3 [7.8, 18.9]	6.5 [4.5, 11.2]	0.007
Bloody nipple discharge				<0.001				0.368				0.119
Absent	269 (85.67%)	148 (93.08%)	121 (78.06%)		28 (71.8%)	13 (81.3%)	15 (65.2%)		45 (72.6%)	28 (80.0%)	17 (63.0%)	
Present	45 (14.33%)	11 (6.92%)	34 (21.94%)		11 (28.2%)	3 (18.8%)	8 (34.8%)		17 (27.4%)	7 (20.0%)	10 (37.0%)	
Quadrant with tumor involvement				<0.001				0.075				0.169
One quadrant involved	229 (72.93%)	135 (84.91%)	94 (60.65%)		29 (74.4%)	14 (87.5%)	15 (65.2%)		35 (56.5%)	23 (65.7%)	12 (44.4%)	
More than one quadrant involved	85 (27.07%)	24 (15.09%)	61 (39.35%)		10 (25.6%)	2 (12.5%)	8 (34.8%)		27 (43.5%)	12 (34.3%)	15 (55.6%)	
Signal intensity on T2 weighted images				0.994				0.786				0.861
Iso and low	240 (76.43%)	121 (76.10%)	119 (76.77%)		31 (79.5%)	13 (81.3%)	18 (78.3%)		49 (79.0%)	28 (80.0%)	21 (77.8%)	
High	74 (23.57%)	38 (23.90%)	36 (23.23%)		8 (20.5%)	3 (18.8%)	5 (21.7%)		13 (21.0%)	7 (20.0%)	6 (22.2%)	
Mass or NME				0.558				0.426				0.465
NME	272 (86.62%)	140 (88.05%)	132 (85.16%)		33 (84.6%)	14 (87.5%)	19 (82.6%)		55 (88.7%)	30 (85.7%)	25 (92.6%)	
Mass	42 (13.38%)	19 (11.95%)	23 (14.84%)		6 (15.4%)	2 (12.5%)	4 (17.4%)		7 (11.3%)	5 (14.3%)	2 (7.4%)	
Enhancement pattern				0.188				0.197				0.218
Homogeneous + clumped	289 (92.04%)	150 (94.34%)	139 (89.68%)		35 (89.7%)	15 (93.8%)	20 (87.0%)		57 (91.9%)	31 (88.6%)	26 (96.3%)	
Heterogeneous + rim	25 (7.96%)	9 (5.66%)	16 (10.32%)		4 (10.3%)	1 (6.3%)	3 (13.0%)		5 (8.1%)	4 (11.4%)	1 (3.7%)	
Intratumor necrosis				0.267								0.664
Absent	299 (95.22%)	154 (96.86%)	145 (93.55%)		37 (94.9%)	15 (93.8%)	22 (95.7%)		59 (95.2%)	33 (94.3%)	26 (96.3%)	
Present	15 (4.78%)	5 (3.14%)	10 (6.45%)		2 (5.1%)	1 (6.3%)	1 (4.3%)		3 (4.8%)	2 (5.7%)	1 (3.7%)	
Ductal ectasia				0.034				0.036				0.035
Absent	219 (69.75%)	120 (75.47%)	99 (63.87%)		26 (66.7%)	13 (81.3%)	13 (56.5%)		41 (66.1%)	27 (77.1%)	14 (51.9%)	
Present	95 (30.25%)	39 (24.53%)	56 (36.13%)		13 (33.3%)	3 (18.8%)	10 (43.5%)		21 (33.9%)	8 (22.9%)	13 (48.1%)	
Nipple‐areolar complex invasion				0.078				0.071				0.079
Absent	273 (86.94%)	144 (90.57%)	129 (83.23%)		34 (87.2%)	15 (93.8%)	19 (82.6%)		54 (87.1%)	33 (94.3%)	21 (77.8%)	
Present	41 (13.06%)	15 (9.43%)	26 (16.77%)		5 (12.8%)	1 (6.3%)	4 (17.4%)		8 (12.9%)	2 (5.7%)	6 (22.2%)	
Initial enhancement pattern of dynamic curve				0.069				0.217				0.177
Slow and medium	38 (12.10%)	25 (15.72%)	13 (8.39%)		5 (12.8%)	1 (6.3%)	4 (17.4%)		6 (9.7%)	2 (5.7%)	4 (14.8%)	
Fast	276 (87.90%)	134 (84.28%)	142 (91.61%)		34 (87.2%)	15 (93.8%)	19 (82.6%)		56 (90.3%)	33 (94.3%)	23 (85.2%)	
Washout during the delayed phase				0.268				0.721				0.724
Absent	179 (57.01%)	96 (60.38%)	83 (53.55%)		20 (51.3%)	8 (50.0%)	12 (52.2%)		34 (54.8%)	18 (51.4%)	16 (59.3%)	
Present	135 (42.99%)	63 (39.62%)	72 (46.45%)		19 (48.7%)	8 (50.0%)	11 (47.8%)		28 (45.2%)	17 (48.6%)	11 (40.7%)	

Abbreviations: ADC, apparent diffusion coefficient; ADCmean, mean ADC; ADCmin, minimum ADC; DCIS, ductal carcinoma in situ; IC, invasive component; NME, non‐mass enhancement.

* *p* value: For variables with a normal distribution, a *t*‐test is used to calculate *p* value for quantitative variables (or Mann–Whitney U test when appropriate), and chi‐square test (or Fisher's exact test when appropriate) is used to calculate *p* value for qualitative variables.

### 3.2 Feature extraction, selection, and radiomics model establishment

From the initially identified 2264 features, 1999 features with ICCs > 0.85 were selected. The data were preprocessed with *Z*‐score normalization, and finally, 13 features were selected using LASSO regression, all of which were texture features. For further specifics on these features, please refer to the Table S3.

Based on the aforementioned 13 features, five models, bagging DT, DT, LR, RF and SVM, were used for radiomics modeling. Among the established radiomics models (Table 2), the bagging DT showed the best overall performance, with an AUC (95% CI) of 0.88 (0.84–0.92) in the training set and 0.78 (0.66‐0.89) in the internal test set, respectively. In addition, the AUC of the bagging DT model in the external test were 0.7 (95% CI: 0.80–0.88) and 0.71 (95% CI: 0.88–0.94) in Center 2 and Center 3 datasets, respectively. Additionally, under this model, the radscore of each patient was obtained.

**TABLE 2:** Diagnostic performance of different machine learning models.

	AUC(95% CI)			
Models	Train (*N* = 251)	Internal test set (*N* = 63)	External test set 1 (*N* = 39)	External test set 2 (*N* = 62)
Bagging decision tree	0.879(0.838–0.919)	0.776(0.662–0.891)	0.700(0.54–0.86)	0.711(0.58–0.84)
Decision tree	0.772(0.715–0.828)	0.798(0.69–0.906)	0.549(0.36–0.73)	0.618(0.48–0.76)
LR	0.751(0.69–0.811)	0.739(0.614–0.863)	0.578(0.39–0.76)	0.703(0.57–0.83)
Random forest	0.889(0.85–0.928)	0.735(0.608–0.863)	0.661(0.49–0.83)	0.696(0.57–0.83)
SVM	0.778(0.722–0.835)	0.738(0.614–0.861)	0.603(0.42–0.78)	0.686(0.56–0.82)

Abbreviations: LR, logistic regression; SVM, support vector machine.

### 3.3 Combined model and radiomics nomogram of construction and evaluation of the three models

**FIGURE 2:**
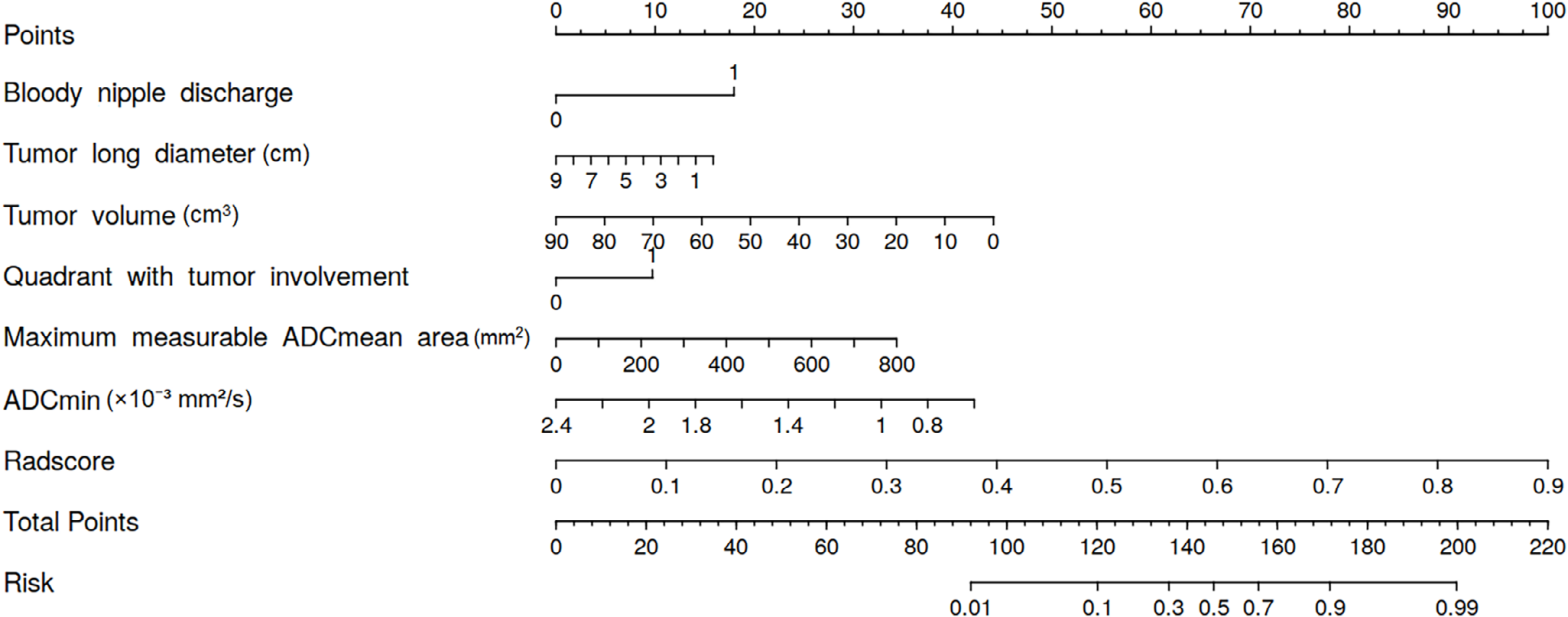
Nomogram was developed in the training set, with the incorporation of bloody nipple discharge, tumor long diameter, tumor volume, quadrant with tumor involvement, maximum measurable ADCmean area, and ADCmin.

The clinico‐radiologic features were combined with the radscore to build a comprehensive combination model. The visualization of the combined model was achieved through the nomogram (Figure 2). The total score was obtained by summing the scores of six factors: bloody nipple discharge, tumor long diameter, tumor volume, quadrant with tumor involvement, maximum measurable ADCmean area, ADCmin, and radscore. The total score corresponded to the probability of infiltrative components in DCIS.

**FIGURE 3:**
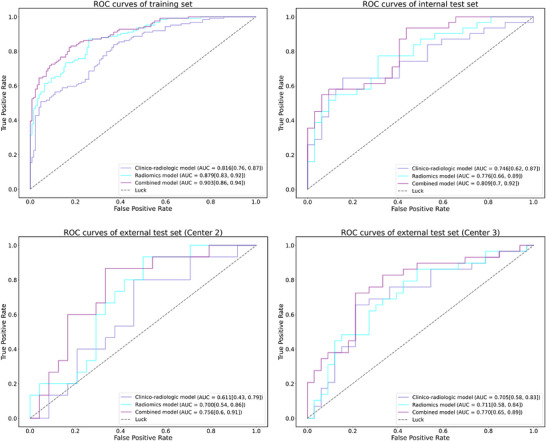
AUC of clinico‐radiologic model, radiomics model, and combined model in the training set, internal test set, and two external test sets. The predictive performance of the combined model for ICs of DCIS was better than that of the clinico‐radiologic model and radiomics model in the training, internal test, and external test sets.

**FIGURE 4:**
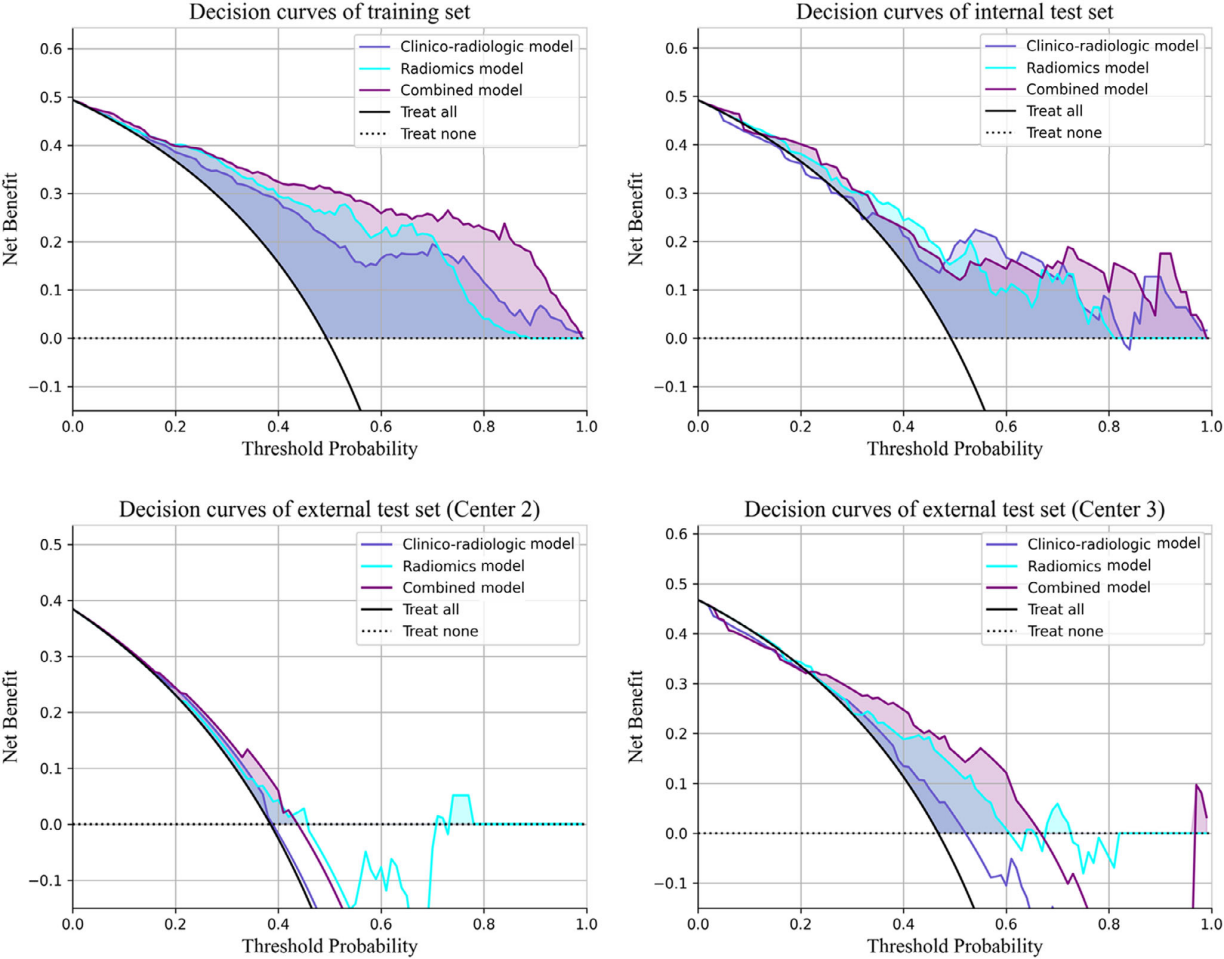
Decision curves analysis (DCA) for three models are shown in the training, internal test, and two external test sets; the y‐axis indicates the net benefit, the x‐axis indicates threshold probability. DCA illustrated that the combined model showed the greatest overall net benefit for upstage within reasonable threshold probabilities.

**FIGURE 5:**
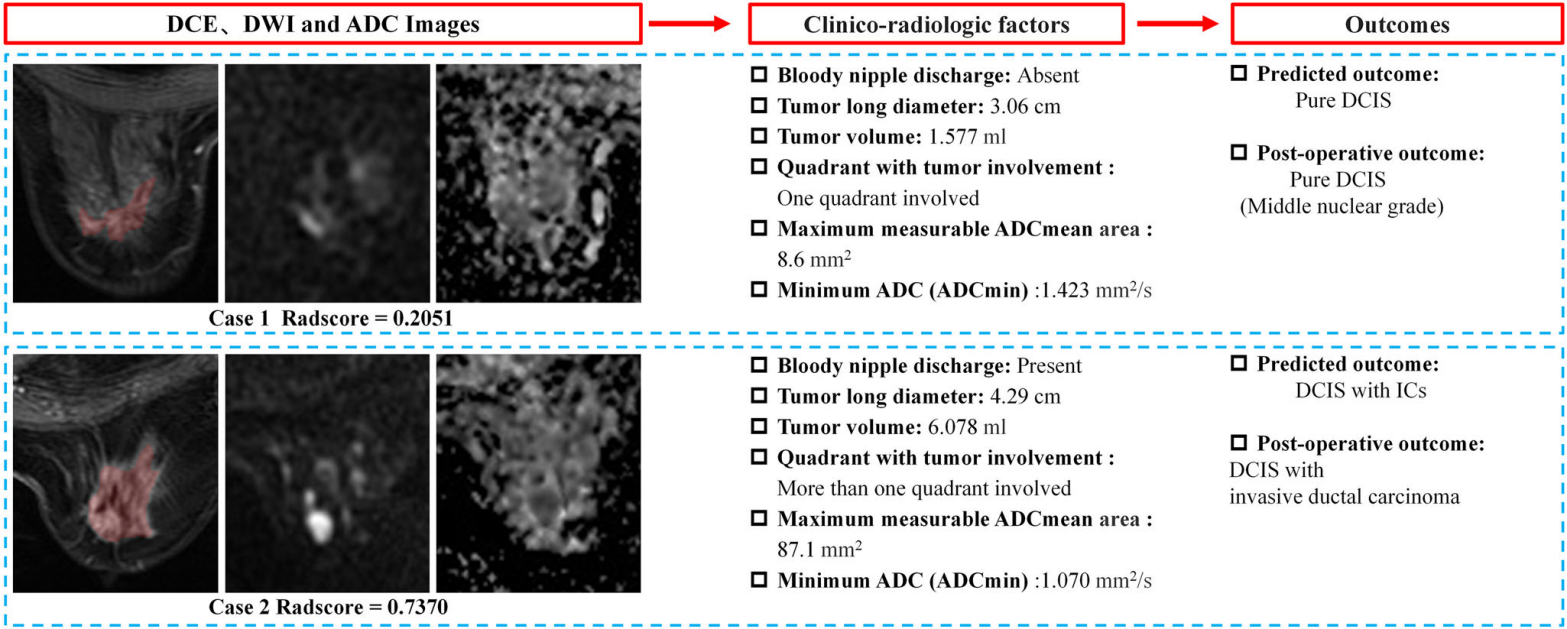
Two representative cases to show discriminative ability of combined model for prediction of ICs. Case 1 predicted pure DCIS and case 2 predicted DCIS with ICs, and the predictions were consistent with the final postoperative pathological findings.

The results presented in Table 3 illustrate the diagnostic performance of individual models and Figure 3 visually shows the AUC for each model. Among them, the AUC values of the combined model were 0.90 (0.86‐0.94) and 0.81 (0.70–0.92) in the training and internal test sets, respectively. The two external test sets had AUCs of 0.76 (0.60–0.91) and 0.77 (0.65–0.89), respectively, which were the highest among the three models. At the optimal cutoff, the sensitivity of the combined model was consistently higher than its specificity in all but the Center 3 dataset, likely reflecting the data heterogeneity introduced by differences in scanners and acquisition protocols. DCA plot (Figure 4) also revealed that the combined model exhibited superior clinical usability. To illustrate the discriminative power of the combined model in predicting ICs, two representative cases are presented (Figure 5). DeLong tests and IDI results (Table 4) indicated that adding the radiomics signature to the clinico‐radiologic model generally led to a significant improvement in prediction. The only exceptions were in the internal testing dataset and the external Center 3 data, where the DeLong test did not reach statistical significance. For further specifics, please refer to Table 4.

**TABLE 3:** Diagnostic performance of the clinico‐radiologic model, the radiomics model, and the combined model.

		AUC(95% CI)		Sensitivity		Specificity		Accuracy	
Internal	Models	Train	Test	Train	Test	Train	Test	Train	Test
Center 1 (*n* = 314)	Clinico‐radiologic model	0.816(0.765–0.872)	0.746(0.621–0.847)	0.839	0.645	0.630	0.844	0.733	0.746
	Radiomics model	0.879(0.831–0.919)	0.776(0.654–0.872)	0.871	0.774	0.740	0.687	0.805	0.730
	Combined model	0.903(0.862‐0.946)	0.809(0.691–0.897)	0.847	0.935	0.803	0.562	0.821	0.746

External		Test			
Center 2 (*n* = 39)	Clinico‐radiologic model	0.611(0.442–0.763)	0.800	0.542	0.615
	Radiomics model	0.700(0.532–0.836)	0.933	0.500	0.667
	Combined model	0.756(0.592–0.879)	0.867	0.667	0.718
Center 3 (*n* = 62)	Clinico‐radiologic model	0.705(0.576–0.814)	0.655	0.788	0.710
	Radiomics model	0.711(0.581–0.819)	0.862	0.515	0.661
	Combined model	0.770(0.646–0.867)	0.724	0.788	0.758

Abbreviations: AUC, area under the receiver operating characteristic curve; CI, confidence interval.

**TABLE 4:** Results of Delong test and IDI for comparison of the diagnostic performance between models.

		Delong		IDI	
Internal	Models	Train	Test	Train	Test
Center 1 (*n* = 314)	Combined model versus radiomics model	*p* < 0.0001	*p* = 0.2782	0.1971, *p* < 0.01	0.1561, *p* < 0.01
	Combined model versus clinico‐radiologic model	*p* = 0.0402	*p* = 0.1997	0.1768, *p* < 0.01	0.1091, *p* = 0.0207
**External**		Test		Test	
Center 2 (*n* = 39)	Combined model versus radiomics model	*p* = 0.5325		0.1128, *p* < 0.01	
	Combined model versus clinico‐radiologic model	*p* = 0.0267		0.0939, *p* = 0.0354	
Center 3 (*n* = 62)	Combined model versus radiomics model	*p* = 0.1785		0.1821, *p* < 0.01	
	Combined model versus clinico‐radiologic model	*p* = 0.1680		0.1651, *p* < 0.01	

Abbreviation: IDI, integrated discrimination improvement.

## 4 DISCUSSION

In this multicenter study, we developed and externally validated a combined model integrating clinical factors, conventional MRI features, and DCE‐MRI radiomics to preoperatively predict invasive upstaging in biopsy‐proven DCIS. The combined model can identify more than 72.4% of surgically confirmed upstaging cases, with a specificity of up to 78.8%, demonstrated superior discrimination (AUCs 0.76–0.90) and net clinical benefit across internal and two independent external test sets. To our knowledge, this is the largest radiomics study to date addressing DCIS upgrade, and the first to incorporate both quantitative DWI parameters and rigorous external validation. Our findings suggest that radiomics can complement conventional MRI to improve risk stratification and inform surgical planning, including the need for sentinel lymph node biopsy.

The ability to accurately determine preoperatively whether DCIS contains ICs is crucial for guiding the subsequent treatment strategy for a patient.^36^, ^37^ Specifically, it affects whether the patient can be put on active surveillance or requires surgical excision and whether a sentinel lymph node biopsy is needed, as patients have different prognoses.^38^, ^39^, ^40^ According to a significant meta‐analysis, an average of 25.9% of DCIS had ICs,^41^ and a similar average rate of 20.9% was reported in another review.^32^ The upstaging rate of DCIS was 26.6% in a very recent review.^42^ In contrast, in this study, we observed a higher upgrade rate, close to 50%, which is comparable to the upgrade rates reported in similar MRI‐based studies.^17^, ^32^, ^43^ The possible reasons for this discrepancy are as follows; first, patients who are enrolled in the study were required to have undergone an MRI examination, whereas, in our organization, more pure DCIS detected by mammography screening is directly resected under mammographic guidance without MRI examination. In other words, patients who have had MRI examinations tend to have lesions that are perceived as more concerning and thus more likely to be upgraded. As previously reported, invasive cancers were more likely to be identified at MRI.^44^, ^45^ Second, most of the specimens analyzed were obtained through core‐needle biopsy, which may miss occult invasive components due to the smaller volume of tissue it samples, compared with vacuum‐assisted biopsy, which is consistent with previous findings.^18^, ^21^, ^32^ Thirdly, it is worth noting that 32 patients were excluded from our study owing to the absence of MRI assessment, all of whom had pure DCIS, which also contributed to reduced upgrade rate.

Our study differs from previous reports in several important aspects. Firstly, although previous single‐center studies^29^, ^30^ achieved comparable predictive performance for invasive components in DCIS, our work incorporated both conventional quantitative MRI parameters and radiomics analysis within a multi‐center framework. Previous radiomics studies largely ignored conventional diffusion‐weighted imaging (DWI) features, whereas in our study, the maximum measurable ADC_mean area and ADC_min were included as predictors. These findings confirmed that quantitative DWI metrics can contribute valuable complementary information in predicting whether DCIS contains invasive components, consistent with earlier research.^17^, ^32^, ^46^ On the one hand, larger maximum measurable ADCmean areas are more likely to contain ICs. This rather intriguing result might be explained by the fact that DCIS lesions typically appear as punctate lesions, which are interspersed with normal tissue, similar to a sponge. Invasive cancers usually appear as irregular masses due to the infiltrative destruction of surrounding tissues, resulting in the merging of multiple foci into a single measurable lesion. On the other hand, lower ADCmin predicts that DCIS is more likely to contain ICs. Because studies have shown that invasive ductal carcinoma has a smaller ADC value than DCIS,^47^, ^48^ it may be that the area displaying the ADCmin could correspond to regions with the highest cell density, the potential nesting sites for DCIS invasion. The lower this value of the region of ADCmin, the more likely it represents an invasive carcinoma. Second, our study developed five machine learning models to predict DCIS upgrade based on thousands of radiomics features on MRI, whereas previous studies were based on a relatively small number of MRI features^31^ or only one machine learning approach.^29^, ^30^, ^49^ This prevents us from getting a full picture of the application. Third, to our knowledge, this study represents the most extensive sample size of related studies to date based on MRI radiomics features and verified using two external validation sets. This shows that the model has good robustness, or what we call generalizability.

The findings of the study revealed that all texture features were selected by the LASSO method for subsequent model construction. LASSO regression, by introducing a regularization term during feature selection, effectively eliminates redundant variables and mitigates overfitting, thereby enhancing the model's robustness and generalizability. This result highlights the superior predictive power of texture features over first‐order or shape features in identifying DCIS with ICs, consistent with previous research findings.^31^ Other studies have confirmed the importance of texture features and the selection of texture features for high frequency.^50^, ^51^ Textural features may reflect the inherent heterogeneity of the tumor. Compared with DCIS, invasive ductal carcinoma of the breast is more pathologically heterogeneous because it consists of cancer cells with distinct morphologies, stroma, fibrillar component, necrosis, and intraductal components.

Our study extracted radiomics features solely from the intra‐tumoral region, as defined on DCE‐MRI. However, given that invasive components may extend into the peritumoral stroma, future work could explore the inclusion of peritumoral radiomics features, which have shown promise in other cancers for capturing microenvironmental heterogeneity.^52^, ^53^ Additionally, although not evaluated here, integrating mammography or digital breast tomosynthesis features may provide complementary information, particularly for detecting calcifications associated with invasion.^54^ Such multimodal approaches could further enhance predictive performance and are a direction for subsequent research.

Notably, this study had some limitations. First, as it was a retrospective study, potential selection bias may hinder the reproducibility and comparability of the results, and the clinical utility of this computational approach still requires further improvement and independent validation. Second, although multi‐parametric MRI has become an important part of breast cancer assessment, only dynamic enhancement sequences were selected in this study. There is no clear consensus on the usefulness of T2‐weighted imaging and DWI in predicting whether DCIS contains ICs, necessitating further studies in this aspect. Third, the radiomics model was entirely based on texture features, which are known to be sensitive to image acquisition and reconstruction parameters and may lack direct biological interpretability. Nevertheless, these features were adopted due to their demonstrated predictive value and consistent performance across multiple validation cohorts. Finally, the sample size was limited; subsequent research should incorporate a larger patient cohort to optimize and validate the precision of this predictive model.

## 5 CONCLUSION

In conclusion, this preliminary study indicates that the combined model based on clinical, conventional MRI, and radiomics features is useful for preoperative prediction of surgically confirmed upstaging in DCIS. As a non‐invasive and quantitative approach, radiomics can be a useful complementary tool to traditional MRI in the clinical decision‐making process.

## AUTHOR CONTRIBUTIONS

**Xi Hu**: Investigation; formal analysis; data curation; visualization; writing—original draft; writing—reviewing and editing. **Lujie Qian**: Investigation; formal analysis; data curation; visualization; writing—original draft; writing—reviewing and editing. **Jie He**: Investigation; writing—original draft;data curation; validation. **Beili Shou**: Investigation; data curation; validation. **Ying Hu**: Investigation; data curation; validation. **Qingqing Chen**: Investigation; data curation; validation. **Enhui Xin**: Investigation; data curation. **Fei Li**: Investigation; data curation. **Zongyu Xie**: Investigation; data curation; validation. **Yue Qian**: Investigation; data curation. **Feifei Lou**: Investigation; data curation. **Nan Liu**: Investigation; data curation. **Yu Kuang**: Conceptualization; methodology; resources; investigation; data curation; writing—reviewing and editing; supervision; project administration. **Hongjie Hu**: Conceptualization; methodology; resources; investigation; data curation; writing—reviewing and editing; supervision; project administration. All authors read and approved the final manuscript.

## CONFLICT OF INTEREST STATEMENT

The authors declare no conflicts of interest.

## ETHICAL APPROVAL

This retrospective study was approved by the Ethics Committee of Sir Run Run Shaw Hospital, Zhejiang University School of Medicine (2024 Research No. 0181), The First Affiliated Hospital of Bengbu Medical University (2025 Research No. 524), and The First Affiliated Hospital of Zhengzhou University (No. 2021‐KY‐0269‐002), the need for informed consent was waived.

## Supporting information




**Supporting Information**: acm270637‐supp‐0001‐TableS1‐S3.docx

## Data Availability

The data that support the findings of this study are available from the corresponding author upon reasonable request. The data are not publicly available due to ethical restrictions and patient confidentiality.
