# Supplementary material for: Multicenter development and external validation of clinical–radiomics models to predict surgically confirmed upstaging in biopsy‐proven DCIS using DCE‐MRI
Source: J Appl Clin Med Phys. 2026 May 31;27(5):e70637. doi: 10.1002/acm2.70637 (PMC13239664; doi:10.1002/acm2.70637)
Supplement: Supplementary file 1 — Supporting Information: acm270637‐supp‐0001‐TableS1‐S3.docx [file ACM2-27-e70637-s001.docx]

### Supporting Material

##### Supplementary Methods

**MRI Data Acquisition**

For the Sir Run Run Shaw hospital center, all MRI scans were performed on a 1.5 T scanner (Signa HD excite, GE Healthcare, Milwaukee, WI, USA). Before the contrast medium was administered, axial DWI images were acquired using two b values (0 and 800 s/mm^2^). A pre-scan of fat-suppressed T_1_WI was performed before scanning fat-suppressed multi-phase dynamic enhanced T_1_WI, and they were acquired as seven post-contrast scans at intervals of 70 seconds following the injection of gadolinium-based contrast agent. A gadolinium-based agent (Gd-DTPA; Beijing Beilu Pharmaceutical Co., Ltd., Beijing, China) was injected using a high-pressure syringe at a rate of 3 ml/s and at a dose of 0.1 mmol/kg of body weight, followed by a 20 ml saline flush with a high-pressure syringe at the same flow rate.

For the First Affiliated Hospital of Bengbu Medical College center, all MRI scans were performed on a 3.0 T scanner (Achieva, Philips Healthcare, Best, The Netherlands). Before the contrast medium was administered, axial DWI images were acquired using two b values (0 and 800 s/mm2). A pre-scan of fat-suppressed T_1_WI was performed before scanning fat-suppressed multi-phase dynamic enhanced T_1_WI, and they were acquired as five post-contrast scans at intervals of 60 seconds following the injection of gadolinium-based contrast agent. A gadolinium-based agent (Gd-DTPA; Beijing Beilu Pharmaceutical Co., Ltd., Beijing, China) was injected using a high-pressure syringe at a rate of 3 ml/s and at a dose of 0.1 mmol/kg of body weight, followed by a 20 ml saline flush with a high-pressure syringe at the same flow rate.

For the First Affiliated Hospital of Zhengzhou University center, all MRI scans were performed on a 3.0 T scanner (Verio; Siemens Healthcare, Erlangen, Germany). Before the contrast medium was administered, axial DWI images were acquired using two b values (0 and 800 s/mm^2^). A pre-scan of fat-suppressed T_1_WI was performed before scanning fat-suppressed multi-phase dynamic enhanced T_1_WI, and they were acquired as five post-contrast scans at intervals of 59 seconds following the injection of gadolinium-based contrast agent. A gadolinium-based agent (Gd-DTPA; Kang Chen Pharmaceutical, Guangzhou, China) was injected using a high-pressure syringe at a rate of 2 ml/s and at a dose of 0.1 mmol/kg of body weight, followed by a 20 ml saline flush with a high-pressure syringe at the same flow rate.

**Table S1** MR scanning parameters for the patients

| **Hospital** | **Sequence** | **TR/TE**  **(ms)** | **FOV**  **(mm)** | **Matrix** | **Slice Thickness (mm)** | **Slice Gap**  **(mm)** | **Slices** | **Flip Angle** |
| --- | --- | --- | --- | --- | --- | --- | --- | --- |
| Sir Run Run Shaw hospital | DWI | 4500/73.6 | 350×350 | 128×128 | 4 | 1.0 | 28 | 90 |
|  | DCE-MRI | 4.9/2.2 | 380×380 | 380×240 | 1.8 | 0 | 88 | 15 |
| First Affiliated Hospital of Bengbu Medical College | DWI | 3000/59 | 406×406 | 188×186 | 4.0 | 0.5 | 32 | 90 |
|  | DCE-MRI | 4.6/2.2 | 355×355 | 324×271 | 1.0 | 0 | 150 | 10 |
| First Affiliated Hospital of Zhengzhou University | DWI | 14200/79 | 340×140 | 192×84 | 4.0 | 0.8 | 34 | 90 |
|  | DCE-MRI | 4.7/1.7 | 384×384 | 384×296 | 1.2 | 0 | 128 | 10 |

FOV, field of view; TR, repetition time; TE, echo time; DWI, diffusion weighted imaging; DCE, dynamic contrast-enhancement

**Details of the radiomics features**

Most of the radiomic features were defined according to the Image Biomarker Standardization Initiative (IBSI) guidelines. In total, three categories of radiomic features were extracted from each image, including first-order (18), shape (14), and texture (72) features. Texture features comprised 21 gray-level co-occurrence matrix (GLCM) features, 14 gray-level dependence matrix (GLDM) features, 16 gray-level run length matrix (GLRLM) features, 16 gray-level size zone matrix (GLSZM) features, and 5 neighborhood gray-tone difference matrix (NGTDM) features. To augment the feature set, 24 image filters were applied prior to feature extraction, including: BoxMean, AdditiveGaussianNoise, BinomialBlurImage, CurvatureFlow, BoxSigmaImage, Laplacian of Gaussian (LoG) (with four sigma values), wavelet decomposition (eight wavelets), Normalize, LaplacianSharpening, DiscreteGaussian, Mean, SpeckleNoise, RecursiveGaussian, and ShotNoise. Based on filtered images, an additional 432 first-order features and 1728 texture features were obtained; shape features were extracted exclusively from the original images. Thus, a total of 2264 radiomic features were originally derived for each ROI.

##### Supplementary Results

**Table S2** Clinico-radiologic features with raw p-values and FDR-adjusted q-values (Benjamini–Hochberg correction)

| **Clinico-radiologic features** | **Raw p-value** | **Adjusted q-value** | **Significant (FDR = 0.05)** |
| --- | --- | --- | --- |
| Age (year) | 0.145 | 0.2054 | FALSE |
| Tumor long diameters (cm) | <0.001 | 0.0024 | TRUE |
| Tumor volume (cm^2^) | <0.001 | 0.0024 | TRUE |
| ADCmean (×10^-3^mm^2^/s) | <0.001 | 0.0064 | TRUE |
| Maximum measurable ADCmean area (mm^2^) | <0.001 | 0.0024 | TRUE |
| ADCmin (×10^-3^mm^2^/s) | <0.001 | 0.0024 | TRUE |
| Maximum measurable ADCmin area (mm^2^) | <0.001 | 0.0024 | TRUE |
| Bloody nipple discharge | <0.001 | 0.0024 | TRUE |
| Quadrant with tumor involvement | <0.001 | 0.0024 | TRUE |
| Signal intensity on T2 weighted images | 0.994 | 0.994 | FALSE |
| Mass or NME | 0.558 | 0.5929 | FALSE |
| Enhancement pattern | 0.188 | 0.2458 | FALSE |
| Intratumor necrosis | 0.267 | 0.3037 | FALSE |
| Ductal ectasia | 0.034 | 0.0642 | FALSE |
| Nipple-areolar complex invasion | 0.078 | 0.1205 | FALSE |
| Initial enhancement pattern of dynamic curve | 0.069 | 0.1173 | FALSE |
| Washout during the delayed phase | 0.268 | 0.3037 | FALSE |

**Table S3** Coefficient of the feature after lasso select

| **Feature name** | **Coefficient** |
| --- | --- |
| wavelet_glrlm_wavelet-LHH-RunVariance | 0.10680047 |
| curvatureflow_glszm_SizeZoneNonUniformityNormalized | 0.09950357 |
| shotnoise_ngtdm_Complexity | 0.07049283 |
| normalize_glrlm_RunPercentage | 0.06805626 |
| log_glcm_log-sigma-2-0-mm-3D-Correlation | 0.05131739 |
| log_glcm_log-sigma-2-0-mm-3D-Imc2 | 0.04929571 |
| log_glszm_log-sigma-4-0-mm-3D-ZoneEntropy | 0.03708861 |
| laplaciansharpening_glcm_Autocorrelation | 0.03395892 |
| boxsigmaimage_glszm_HighGrayLevelZoneEmphasis | 0.02108146 |
| log_glrlm_log-sigma-4-0-mm-3D-RunEntropy | -0.006068 |
| wavelet_glrlm_wavelet-HHH-RunEntropy | -0.0073493 |
| normalize_glszm_ZonePercentage | -0.0453102 |
| boxsigmaimage_glszm_ZoneEntropy | -0.0619262 |
